# “Quality Women’s Healthcare Should Be A Top Priority”: Service Women’s Experiences With the Health Care Provided by the Netherlands Armed Forces

**DOI:** 10.1093/milmed/usae537

**Published:** 2024-12-05

**Authors:** Mohan K Verstegen, Eva M van Baarle

**Affiliations:** Defense Healthcare Organization, Royal Netherlands Armed Forces, Utrecht 3584AB, The Netherlands; Faculty of Military Sciences, Royal Netherlands Armed Forces, Breda 4811XC, The Netherlands; Department of Medical Humanities, Amsterdam UMC, Location VUmc, VU Amsterdam, Amsterdam 1081 HV, The Netherlands

## Abstract

**Introduction:**

Fit and healthy military personnel are the basis for a strong organization and good health care is essential to ensure service people’s deployability. This applies equally to female-specific health care (FSH). Quality health care can help not only to recruit but also retain more women in the military. However, as there is a lack of empirical studies focusing on service women’s experiences with FSH, this study explores female military personnel’s experiences with FSH in the Netherlands Armed Forces (NAF), including conditions such as menstruation, pregnancy, and menopause in order to find ways of improving these women’s health care and self-care.

**Methods:**

We carried out a qualitative study involving 4 focus groups of service women (*n* = 17) from all military branches of the NAF (Navy, Air Force, Army, and Military Police).

**Results:**

Using inductive thematic analysis, we ascertained 4 key themes: inadequate knowledge and experience among military primary care providers; a perceived lack of gynecologists in the military health care system; difficulties around FSH-related self-care; and inadequate (and inadequately provided) information on menstruation, pregnancy, and menopause. Our findings imply several recommendations to foster good FSH. Changes are needed on four fronts: agenda setting, information, normalization, and implementation. Leaders and role models can bring the topic FSH to the chain of command’s attention.

**Conclusion:**

The NAF are currently not focusing sufficiently on FSH. In order to provide their female personnel with a high quality of care, this will require changes in policy, implementation, and supervision as well as fostering the development of an open culture that enables discussions on FSH.

## INTRODUCTION

In the past decennia, more and more women have joined the military services, though they are still a minority. Military organizations have attempted to improve service women’s social inclusion, by increasing the number of female personnel and by actively seeking to recruit and retain more women in all military ranks.^1^ Adopting policies and practices that accommodate female personnel’s health care needs is regarded as a key element within this strategy.^2^

The Netherlands Armed Forces (NAF) are no exception and aim to have a 30% female workforce (as opposed to 11% in 2021) by 2030.^3^ The NAF provide in-house health care for their personnel focussing on prevention, cure, and occupational health. This includes dental, primary and secondary care, mental health care, and rehabilitation—nearly the entire range of medical disciplines.

In their role as health care provider for their personnel, the NAF are required to ensure they provide good quality health care. This is not only crucial from a preventive and curative perspective but also in terms of occupational health: healthy military personnel simply perform better. Accordingly, the health of all personnel is crucial to the operational readiness of the entire armed forces.^4^ This includes female-specific health care (FSH; Table 1).

**Table 1.** An Overview of Female-Specific Health Care Topics

Self-care	
	Menstruation
	Birth control
	Menopause
	Conception, pregnancy, and postpartum issues
	Personal hygiene and toilet use
Primary and secondary health care (diagnosis and treatment)	
	Amenorrhea
	Vaginal bleeding
	Birth control
	Vaginal discharge
	Miscarriage
	Sexual disorders and sexually transmitted diseases testing
	Subfertility
	Breast exams and pap smears
	Menstruation
	Menopause
	Conception, pregnancy, and postpartum issues

Three factors have been identified in the literature as affecting military FSH: the availability and quality of services and products; self-care options; and a male-dominated culture.^5–8^

Ritchie^5^ has detailed the health care needs of women in the U.S. military. These include improvements in hygiene, prenatal care, cervical screening, information for commanders, and the availability of gynecological and contraceptive care. Manski^6^ reports that the confidentiality of consultations is also a barrier to seeking help and that the lack of female health care providers is an area for improvement. Watkins^7^ concludes that 39% of women firefighters reported that their work was affected by menstrual or menopausal complaints. In a U.S. study, young women nonofficers (aged 20–29 years) reported more FSH issues than women officers.^8^ This established a link between physical and mental stress in the early years of their working life and the occurrence of FSH issues. A recent report on the civilian situation in the Netherlands highlights the relevance of these issues under four headings^9^: their far-reaching effects on health; the associated taboos; quality of life and being part of the work force; and the societal costs.

The above studies provide a starting point for understanding service women’s health care needs and experiences; however, there is scarcely any qualitative exploration of women’s experiences with FSH in the military.^10^ This study presents the findings of qualitative research on service women’s experiences of FSH within the NAF, covering topics such as menstruation, pregnancy, and the menopause. It aims to explore ways of improving health care and self-care in the Armed Forces. The outcomes of this research will contribute to putting FSH on the agenda and provide an impetus for proposed policy and organizational changes.

## METHODS

Given the scarcity of empirical research on service women’s experiences with FSH, we opted for a qualitative and exploratory approach. Focus groups were used as these allow participants to talk in depth and offer opportunities for discussion among peers.^11^ Focus groups were viewed as particularly suitable for understanding aspects of FSH, as they concentrate on the participants’ frames of reference rather than those of the researchers.^12^

### Data Collection

Participants were recruited through posters in military health centres, the Dutch Women’s Defence Network and social media. To ensure that all military branches were represented in the focus groups, an additional call was made for Air Force and Navy service women to take part in the fourth focus group. Table 2 shows the participants’ demographics. Four semistructured focus groups were held with a total of 17 participants between April and October 2023, with each session lasting roughly an hour and a half. Participants were asked a series of open-ended questions about their experiences. The full spectrum of care was considered: self-care, primary care and secondary care. A list of topics served to formulate open-ended questions about the participants’ experiences and they were encouraged to talk to each other. Each focus group was audio recorded and transcribed.

**Table 2.** Participant Demographics

Participants (*n* = 17)		
Age		Between age 28 and 56 years
Operational command	Royal Netherlands Navy	4
	Royal Netherlands Marechaussee/Military Police	3
	Royal Netherlands Army	6
	Royal Netherlands Air Force	4
Rank	Noncommissioned officer (NCO)	5
	Officer (OFF)	12

### Data Analysis

We applied Braun’s thematic analysis, first ascribing initial codes to the data, then shifting to themes.^13^ No pre-existing coding framework was used. A collaborative discussion between the authors followed the open coding, resulting in a tentative overview of themes. The transcripts were coded by the first author. For the final coding, the tentative overview of themes was further specified in collaborative discussions. We used each new transcript to check and validate already recognized themes, and add new themes as they emerged, a saturated description of themes was the final result. The qualitative software program Nvivo was used to record the codes for the analysis and for the process that led to the formulation of themes. All discussions and accompanying decisions were recorded in memos.

## RESULTS

Four main themes emerged from our analysis: first, a lack of knowledge and experience among military primary care providers. Second, a perceived lack of gynecologists in the NAF-health system. Third, the difficulties that many service women experience with self-care. And, fourth, inadequate and inadequately provided information on pregnancy and the menopause.

### Lack of FSH Knowledge and Experience among Military Physicians

Service women perceive a lack of knowledge and experience regarding FSH among military primary care providers, both in terms of their substantive knowledge of FSH and their perceived skill in performing procedures, such as breast and speculum examinations.

“I noticed a significant lack of experience, with women’s health issues in particular.”

This lack of knowledge and experience makes service women feel uncomfortable, misunderstood, not taken seriously, and distrustful of military physicians. These feelings are more pronounced if the doctor is male, younger, or displays certain behaviors such as condescension, insecurity, or trivialization of complaints.

“He was an older man and he came across as really condescending. He said something like: Well, most women find a hot water bottle helps.’’

“And then he concluded: That’s just how it is, and I thought: Well, thank you very much. It can still be a real problem and makes it hard to get on with your job!’’

Women find female physicians are better at listening. They feel that women understand them better and speak the same language.

“I think it’s because of our common frame of reference. Chances are that you’ll both use the same words to describe a specific feeling if you’re discussing breast tightness.”

“Maybe you have had the same complaints and can relate to what the other person is saying. That’s how I see it.’’

“Even when you face an inexperienced woman doctor, she at least understands what you are saying.”

The physician’s age is also significant. Older physicians are often thought to be more experienced. Their FSH experience and communication skills are rated more highly. Some women seem to feel embarrassed about discussing FSH with younger male physicians.

“Sometimes it’s just easier to talk to a woman than to a young chap.’’

A lack of trust and the feeling of not being understood or taken seriously lead to women in the military feeling unappreciated by the organization.

“Quality women’s healthcare should be a top priority for the NAF.”

Service women who have had bad experiences with military physicians indicate that they delay seeking care or forego consultations altogether.

“I walk in and he already has the answer. I don’t even get to tell my story and I think, well, suppose it’s something serious and then if this guy - this doctor - is at the military health centre, I think, you know what? Never mind.”

“The risk is that people will simply give up. And that the threshold gets higher and higher, so people just stop going for pap smears ……. That’s the risk we are running right now. If you don’t keep up with the times, keep running into a brick wall when you request a referral ……. the negative experiences keep piling up and then you either resort to Google or you find a way around it. Or you simply give up.’’

Sometimes service women simply choose to put up with the inconvenience—crossing their own boundaries, in retrospect.

“If you would prefer to be seen by a woman doctor or assistant, you can come back another day - when a woman doctor would be available. It seemed a bit inconvenient at the time, so I told myself to just get over it. I did, but it was definitely awkward. It didn’t go very well.’’

“When I presented with heavy bleeding … he said: ‘I am going to refer you to a specialist, but I’d be interested to take a look anyway.’ And then he said, ‘You don’t mind, do you?’ I say no, of course not, because I’m thinking, don’t be childish. I can’t quite put my finger on it, because he didn’t really do anything wrong. But all the same: I ultimately walked out the door with a kind of bad feeling. I thought, well, was it really necessary for you to take a look? That never happens with a woman - a woman doctor - so now I always ask for a woman.”

Well, I really feel physically uncomfortable when that happens. Really bad. All I heard was something like ‘Can I take a look anyway?’ Something like that. I froze. I couldn’t react. So I automatically just went along with it. Then afterwards, I started thinking. It really affects you personally. It just didn’t add up. But what do you say at that point? We all assume we can speak up, but it’s not that easy. Afterwards, things like this make you think: It doesn’t feel right, what just happened.”

Some women resort to lies to get out of a speculum examination—“‘I’m on my period. Can it wait until next time?’”—or go looking for other physicians, within the NAF or beyond.

Service women indicate that a perception of suboptimal treatment can lead to an increased risk of impaired performance, sick leave, and, in the case of chronic complaints, career delays.

“If the reason for my heavy, irregular periods had been detected and treated earlier, I would have spent much less time off sick.”

In addition to these experiences with treatment by military physicians, another frequently reported issue is the lack of regular, familiar primary care providers for FSH complaints, and the resulting lack of continuity.

Throughout their careers, military personnel are often reassigned to a different barracks every 3 to 5 years. Since care is supposed to be provided at the barracks where they are stationed, military personnel see different physicians all the time. This is exacerbated by the fact that there are too few physicians, especially women physicians. This lack of continuity means that FSH histories inevitably need to be repeated over and over again to each new physician, making it impossible to develop a relationship of trust. Women find this very unpleasant, especially regarding FSH.

“Normally, you build up a rapport with someone over time. It’s kind of strange that the NAF don’t provide for that.”

### The Need for Gynecologists in Secondary Care

The lack of staff gynecologists is considered a major shortcoming. Service women indicate that waiting lists for other specialities at the Central Military Hospital are very short or even nonexistent. Referrals to civilian gynecologists currently almost always result in a long delay, even to gynecologists at the University Medical Centre Utrecht, which has a partnership with the NAF. Military primary care providers seem hesitant to confer with civilian gynecologists: because there is not the same ease as there would be in conferring with NAF gynecologists, the threshold regarding civilian gynecologists is apparently higher. Women also see the risk of a reduced focus on deployability and sick leave. Civilian gynecologists are often unfamiliar with the military and military primary care physicians, and do not seem to assess the gynecological care provided in terms of deployability and sick leave. Repeated referrals for diverse gynecological conditions mean that women end up seeing too many different gynecologists, precluding the development of a relationship of trust. The bottom line is that all these shortcomings make service women feel excluded:

“gynaecological - secondary - care should have equal priority with surgery and dermatology at the NAF.”

The only advantage of this lack of gynecologists is that service personnel are often referred to specialists close to where they live. This advantage is even more pronounced if a patient’s partner needs to be present for a consultation, for example where there are fertility issues.

### Self-care Issues

When service women shared their experiences with self-care, examples of how little their FSH-related needs are taken into account included: Too little time to use the toilet during exercises, often no opportunities to change a tampon. Mobile toilets are often not provided. Menopausal complaints are dismissed as “just par for the course,” there are no facilities for breastfeeding mothers, as there is no policy on postnatal lactation, and uniforms are not designed for women’s bodies. The women felt a lack of consideration for them as individuals and a general lack of consideration for women in the military. These experiences can also lead to tacit acceptance of the situation “we work in a male-dominated profession, nothing we can do about it” and to mishaps (menstrual or urine leaks) experienced as embarrassing.

“Coats should be functional, but a coat that is way too roomy at the top ceases to be functional. Coats should simply fit, at the top and at the bottom. If you need a bigger coat, the openings at the top will be bigger too. It means that cold, rain, wind —- everything just gets in. Just because you need a larger size to accommodate your hips. And the sleeves are about 10 cm too long, so you have to roll them up. Well, it’s simply not practical.”

“I once had a horrible row about having to wear that skirt and those court shoes. Just plain weird.”

“At one point I mentioned: I do need to express milk. Well, nobody had given that any thought. That there actually had to be a place where I could go to express milk. It ended up being the broom cupboard.”

“Speaking of expressing. I had to resort to doing it in the office. One day a guy even came in and asked if he could sit there. I don’t think so. He was a bit startled, of course. But then I thought, I even put up a note on the door. No arrangements were made, though they had nine months to do it.”

This lack of consideration also causes stress and anger. Additional stress is particularly undesirable during training.

“Oh yeah, I still need to change my tampon. Then you forget and it starts leaking. Is that stressful? Yes, it’s annoying and it makes you feel dirty, and of course causes additional stress.”

“During training you are much more vulnerable. You have to stay on good terms with the instructor because that’s the person who decides whether you make the cut.”

You’re already under pressure. So the additional stress—of needing to go to the toilet—doesn’t help. It just makes things worse. I do think it has a negative effect on your performance.”

Two underlying mechanisms have been identified as explaining this insufficient consideration. First, there is a lack of understanding of FSH issues among male military personnel and within organizations. This is due in part to a lack of knowledge. There is also a perception that it is embarrassing to talk about these issues.

Second, the urge to fit in with “the men,” the urge to conform to the masculine norm of perseverance and toughness, and fear of ridicule makes it almost impossible to speak up about FSH needs.

“I just find it all a bit difficult. At the Royal Military Academy, for example, I always had the feeling that I was being made out for a fool. Because I went to the toilet and it made me a little bit late for something. So I explained that it takes a bit longer when you are on your period. It’s not like they were shouting it out in front of the whole group, but everyone could still hear it. So you’re made to look a bit stupid.”

“Things like that are annoying in the workplace and people are awkward about it.”

“You just want to be appreciated as a member of the military.”

To say that women’s needs cannot always be accommodated because “it won’t be possible on deployment either” is seen as a false counter-argument: our participants indicate that allowances can be made to accommodate women in almost any deployment situation, unless there is imminent danger. It should anyway be possible to accommodate women during exercises:

“I think you should just be able to have your period during exercises without having to adjust your menstrual cycle in order to manoeuvre around some S3 exercise planner’s schedule.”

It has also been suggested that some women occasionally take advantage of the rules. For example, by taking extra showers during exercises just because it feels nice, not because it is necessary as a result of menstruation. Consequently, women are sometimes reluctant to avail themselves of certain opportunities: they fear the scorn of their male colleagues.

“Suppose accommodations are made and you are given a bit more time or something like that. The problem then is raised eyebrows in the rest of the group. And that bothers me because it affects your status within the group. That’s also difficult.”

Women are constantly on the lookout for creative solutions:

“I was just in the toilet and saw one of my corporals with two of those cups. She was standing there - expressing breast milk - with her gear on the shelf by the mirror.”

Delaying urination and not changing tampons on time can lead to serious health issues. Some service women even take the pill continuously to skip their period during an exercise.

“Why should I have to adapt my menstrual cycle to suit my employer? Hell no!’’

And finally, some of the women pointed out that the menopause, for example, does not affect everyone the same way. This could explain why some women feel that no changes are needed.

### Inadequate Information (Provided) on Pregnancy and Menopause

Participants argue that insufficient information is provided about pregnancy and menopause at different levels. For pregnancy-related care, servicewomen are sent to the civilian care system. Participants argue that there is a lack of medical advice from military health care professionals, including colleagues as well as commanding officers. This was often mentioned during discussions on menstruation, pregnancy, and the menopause.

There is a clear need for more menopause counsellors throughout the NAF. Individual service women who are going through the menopause require expert, tailored advice to alleviate their symptoms and improve their performance. Many women indicate that it is helpful to talk about it with colleagues. The specific military context poses additional challenges.

“It’s that mix that makes it so difficult to get on with what we’re doing. It’s not just office work, it’s also physical labour.”

Primary health care can be improved by increasing the level of expertise. All personnel should be better informed about, for example, the symptoms and consequences of the menopause. Better knowledge can help create better understanding. Finally, superior officers should be better informed about pregnancy policies and services.

Tackling the key issues identified and improving care for service women require a strategy that includes at least the following steps: agenda-setting, normalization, information, and implementation.

#### Agenda-setting

FSH, and why it is important for the organization, should be high on the agenda. It is up to military personnel, especially military leaders, to make that case. Leaders and ambassadors who champion this issue should be given a platform.

#### Information

The availability of information about FSH issues and how to address them can and must be improved at all levels, for service women, their male colleagues, primary care providers, superior officers, and the organization as a whole.

#### Normalization

Putting FSH issues on the agenda and providing information can improve our understanding and normalize issues such as tampon changes and hot flushes. This should make it easier for people to talk about any issues that arise. The Armed Forces are not necessarily accustomed to this kind of open discussion. Lines of communication need to be kept open. A safe environment for discussion is a prerequisite—without fear of ridicule or repercussions during training.

#### Implementation

Many of these measures are relatively easy to implement: barracks should have facilities for breastfeeding mothers, and women should have well-fitting and functional clothing.

## DISCUSSION

This study explores service women’s current experiences with FSH and how they think it can be improved. The analysis identifies 4 key FSH-related themes: inadequate knowledge and experience among military primary care providers; the need for gynecological care organized by the military, self-care issues, information, and the availability thereof.

Our findings identify 4 strategies for improving FSH: agenda-setting, information, normalization, and implementation.

Self-care issues, the need for gynecological care, and the availability of information are all issues identified by service women in the Netherlands. Similar issues have been reported within the U.S. Army.^5,6^

Our findings show that the NAF should pay more attention to their female personnel’s specific care needs. While future work could explore the generalizability of our findings, we know that individual service women often adapt to the prevailing masculine norms and culture, for instance by concealing their femininity, and rely on support networks for and by military women.^14,15^ Rather than shifting the responsibility to the women, this also calls for action on an organizational level.^16^

For instance, it should be possible to make a primary care appointment with a woman physician. This can also be arranged at the regional level. Expertise and experience are other important factors. One option is to train several primary care providers to become experts in this field. Another option is to use continuing education to promote this specific expertise among military physicians. The advantage of the first option is that these providers would have enough patients with FSH complaints to keep their skills up to date. In any case, women should not have to see a different health provider each time they present with women’s health issues.

Accordingly, our recommendation is to resume hiring staff gynecologists. In addition to regular specialist care as normally provided within the organization, gynecologists could also help build expertise among primary care providers and advise Human Resources (e.g., on pregnancy matters).

Our findings indicate that there is a clear need for menopause counsellors. This could be a way to improve women’s health care at the individual level. As well as advising superior officers and policymakers, they can build expertise among military primary care providers and provide personalized health care recommendations.

We recommend that these measures are embedded in policy to ensure they are structurally maintained and funded. This is also where targets and desirable outcomes should be established, including efforts to put FSH on the agenda by incorporating it into training and leadership programmes. Organizational changes can be implemented, such as increasing the number of women military physicians and menopause counsellors.

## CONCLUDING REMARKS

Much remains to be done to improve the quality of FSH and ensure that service women are able to look after their own health. Good health care provision is not only a prerequisite in order to prevent service women’s avoidance and postponement of seeking care. It is also an expression of a focus on and recognition of women within the Armed Forces and to maintain the operational readiness of the entire Armed Forces. It can help women feel more seen and heard, valued, and included. FSH requires specific measures, especially in organizations such as the NAF, where individual women often adjust to dominant masculine norms. It requires extra effort and embedding. All of this is necessary to recruit and retain more women in the military. The NAF have set this target, and it is now incumbent upon them to act.

## Data Availability

The datasets analyzed during the current study are available from the corresponding author on reasonable request.

## INSTITUTIONAL CLEARANCE

The approval was given by the Military Healthcare Authority.
